# EMERGENT PRESENTATION OF AN OOCYTE RETRIEVAL COMPLICATION

**DOI:** 10.51894/001c.123131

**Published:** 2024-08-31

**Authors:** Garrett Tomaski

**Affiliations:** 1 Emergency Medicine Henry Ford Jackson

## 98

### INTRODUCTION

In vitro fertilization (IVF) is the most common type of assisted reproductive technology (ART). IVF success rates depend on many factors (a woman’s age, use of fresh or frozen eggs) and numbers vary according to ART clinic. For the woman, IVF involves a series of hormone injections (eg, FSH, LH) to induce ovulation and results in a group of mature ovarian eggs. After this 2-week induction, oocyte retrieval is performed via transvaginal ultrasound (US)-guided needle aspiration. Potential complications of oocyte retrieval include ovarian hemorrhage, damage to organs such as the uterus, bladder, intestinal tract, and large blood vessels, and pelvic infection. Our case involves an emergent presentation of an oocyte retrieval complication. The case demonstrates the usefulness of point of care ultrasound (POCUS) in the IVF patient with an acute abdomen.

### CASE DESCRIPTION

A 25-year-old female presented to the Emergency Department (ED) with generalized abdominal pain. Her pain began 4 hours after a bilateral transvaginal oocyte retrieval at an outside hospital. While waiting in the ED lobby, she also had a brief syncopal episode. Vitals were remarkable for tachycardia (124 bpm) and hypotension (95/65 mmHg). On exam, she was in good spirits and no apparent distress. Although her abdomen was soft and non- distended, she had generalized tenderness to palpation and peritonitis elicited on heel strike and bumping the bed. Given the patient’s vitals and exam, they prompted a POCUS FAST, which revealed a large amount of intraperitoneal hypoechoic fluid. Decision was made to admit the patient at our facility after Ob/Gyn consult and trend the patient’s hemoglobin every 4 hours. The Hgb trended downward over night 11.1 >7.8 g/dL and the patient was transferred the next morning. A CTA of abdomen/pelvis was done at outside facility showed a large volume hemoperitoneum with a blush of contrast posterior to the right ovary and within the left adnexa, concerning for arterial extravasation. Complications during IVF are generally infrequent, the frequency of hemoperitoneum after oocyte retrieval has been reported to be 0.06-0.35%, data on the development of OHSS is limited, but generally ranges from 0.14-1%. Patient next day underwent laparoscopic evacuation of hemoperitoneum and discharged the next day in good condition.

